# The effect of dietary nitrate supplementation on the spatial heterogeneity of quadriceps deoxygenation during heavy‐intensity cycling

**DOI:** 10.14814/phy2.13340

**Published:** 2017-07-25

**Authors:** Brynmor C. Breese, David C. Poole, Dai Okushima, Stephen J. Bailey, Andrew M. Jones, Narihiko Kondo, Tatsuro Amano, Shunsaku Koga

**Affiliations:** ^1^ School of Biomedical and Healthcare Sciences Plymouth University Plymouth United Kingdom; ^2^ Departments of Kinesiology and Anatomy and Physiology Kansas State University Manhattan Kansas; ^3^ Applied Physiology Laboratory Kobe Design University Kobe Hyogo Japan; ^4^ School of Sport Exercise and Health Sciences Loughborough University Loughborough United Kingdom; ^5^ Sport and Health Sciences College of Life and Environmental Sciences University of Exeter Exeter United Kingdom; ^6^ Faculty of Global Human Sciences Kobe University Kobe Japan; ^7^ Faculty of Education Niigata University Niigata Japan

**Keywords:** Heterogeneity, nitrate supplementation, oxygen delivery, oxygen utilization, time resolved near‐infrared spectroscopy

## Abstract

This study investigated the influence of dietary inorganic nitrate (NO_3_^−^) supplementation on pulmonary O_2_ uptake ($\dot{V}$O_2_) and muscle deoxyhemoglobin/myoglobin (i.e. deoxy [Hb + Mb]) kinetics during submaximal cycling exercise. In a randomized, placebo‐controlled, cross‐over study, eight healthy and physically active male subjects completed two step cycle tests at a work rate equivalent to 50% of the difference between the gas exchange threshold and peak $\dot{V}$O_2_ over separate 4‐day supplementation periods with NO_3_^−^‐rich (BR; providing 8.4 mmol NO_3_^−^∙day^−1^) and NO_3_^−^‐depleted (placebo; PLA) beetroot juice. Pulmonary $\dot{V}$O_2_ was measured breath‐by‐breath and time‐resolved near‐infrared spectroscopy was utilized to quantify absolute deoxy [Hb + Mb] and total [Hb + Mb] within the *rectus femoris*,* vastus lateralis*, and *vastus medialis*. There were no significant differences (*P *>* *0.05) in the primary deoxy [Hb + Mb] mean response time or amplitude between the PLA and BR trials at each muscle site. BR significantly increased the mean (three‐site) end‐exercise deoxy [Hb + Mb] (PLA: 91 ± 9 vs. BR: 95 ± 12 *μ*mol/L, *P *<* *0.05), with a tendency to increase the mean (three‐site) area under the curve for total [Hb + Mb] responses (PLA: 3650 ± 1188 vs. BR: 4467 ± 1315 *μ*mol/L sec^−1^, *P *=* *0.08). The $\dot{V}$O_2_ slow component reduction after BR supplementation (PLA: 0.27 ± 0.07 vs. BR: 0.23 ± 0.08 L min^−1^, *P *=* *0.07) correlated inversely with the mean increases in deoxy [Hb + Mb] and total [Hb + Mb] across the three muscle regions (*r* ^2^ = 0.62 and 0.66, *P *<* *0.05). Dietary NO_3_^−^ supplementation increased O_2_ diffusive conductance across locomotor muscles in association with improved $\dot{V}$O_2_ dynamics during heavy‐intensity cycling transitions.

## Introduction

Nitric oxide (NO) is a gaseous signaling molecule widely believed to play a crucial role in regulating vascular function and mitochondrial respiration (Brown 2001; Moncada and Higgs 2006). The most recognized pathway for NO production is the five‐electron oxidation of L‐arginine in a reaction catalyzed by the nitric oxide synthase (NOS) enzymes (Bredt et al. 1991). More recently, an alternative O_2_‐independent pathway has been identified where NO is produced from the stepwise reduction in inorganic nitrate (NO_3_^−^) to nitrite (NO_2_^−^) and subsequently NO (Lundberg and Weitzberg 2009). Previous studies have reported that dietary supplementation with sodium nitrate or NO_3_^−^‐rich beetroot juice (BR) elevates plasma [NO_2_^−^] (and therefore the potential for O_2_‐independent NO production; Lundberg and Weitzberg 2009), increases skeletal muscle blood flow (Ferguson et al. 2013b), lowers exercise mean arterial pressure (Ferguson et al. 2013b; Bond et al. 2014) (hence vascular conductance is improved; Ferguson et al. 2013b), slows the reduction in microvascular O_2_ partial pressure (*P*O_2_) (Ferguson et al. 2013a), and reduces O_2_ uptake ($\dot{V}$O_2_) (Larsen et al. 2007, 2010, 2011; Bailey et al. 2009, 2010; Vanhatalo et al. 2010; Lansley et al. 2011; Cermak et al. 2012; Wylie et al. 2013) during skeletal muscle contractions. Therefore, potentiating the NO_3_^−^→NO_2_^−^→NO pathway through dietary NO_3_^−^ supplementation has the potential to improve the matching between muscle O_2_ delivery ($\dot{Q}$O_2_) and metabolic demand ($\dot{V}$O_2_), particularly in the contracting skeletal muscles where the acidic and hypoxic milieu might compromise NOS‐derived NO, but potentiate NO_2_^−^‐derived NO production.

Muscle deoxyhemoglobin/myoglobin (i.e. deoxy [Hb + Mb]) measured by near‐infrared spectroscopy (NIRS) is considered to provide an index of local O_2_ extraction (Koga et al. 2012) and hence to reflect the dynamic (im)balance between $\dot{Q}$O_2_ to $\dot{V}$O_2_ within myocytes and microvessels (DeLorey et al. 2003; Grassi et al. 2003). A previous study has reported that 6 days of BR supplementation decreased the primary deoxy [Hb + Mb] amplitude (i.e. consistent with an increased $\dot{Q}$O_2_/$\dot{V}$O_2_ ratio) during step cycling from unloaded pedaling to a moderate‐intensity work rate (Bailey et al. 2009). Conversely, no effect following BR intake has been reported on muscle deoxy [Hb + Mb] kinetics during step cycling in normoxia from unloaded pedaling to a severe‐intensity work rate (Bailey et al. 2009; Kelly et al. 2014), whereas BR has been reported to speed the time constant (*τ*) of primary deoxy [Hb + Mb] and $\dot{V}$O_2_ kinetics (i.e. muscle O_2_ utilization was enhanced) during step cycling from a moderate‐ to a severe‐intensity work rate (Breese et al. 2013). An important limitation from previous studies using continuous wave (CW‐) NIRS (Bailey et al. 2009, 2015; Kelly et al. 2014) is the assumption of constant tissue optical properties (i.e. path length, absorption and scattering coefficients) which has been reported to confound interpretation of muscle deoxy [Hb + Mb] responses during exercise (Ferreira et al. 2007). Therefore, time‐resolved (TRS‐) NIRS, which permits continuous measurement of the tissue optical properties to resolve absolute deoxy [Hb + Mb] data (Koga et al. 2011), would improve mechanistic insight into BR effects on muscle QO_2_‐to‐VO_2_ relationships.

It is also presently unclear whether dietary NO_3_^−^ supplementation might alter the muscle spatial heterogeneity in deoxy [Hb + Mb] kinetics (and, by extension, $\dot{Q}$O_2_‐to‐$\dot{V}$O_2_ matching) which has previously been reported during cycling exercise (Koga et al. 2007, 2011; Chin et al. 2011; Bowen et al. 2013; Spencer et al. 2014; Fukuoka et al. 2015; Okushima et al. 2015, 2016). Given that NO_2_^−^ reduction to NO is potentiated in low O_2_ environments (Lundberg and Weitzberg 2009), and that NO_2_^−^itself can promote vasodilation in hypoxic tissues (Gladwin et al. 2000), NO_3_^−^ treatment (and the associated increase in circulating plasma [NO_2_^−^]) may be particularly effective at improving vascular function within muscle regions where a low $\dot{Q}$O_2_/$\dot{V}$O_2_ ratio is manifest, for example, within type II muscle or muscle fibers (see (Jones et al. 2016) for review). As well, an increased NO bioavailability via its effects on competing with O_2_ for the O_2_‐binding site at cytochrome‐*c* oxidase (COX) in the electron transport chain (Brown 2001) may inhibit respiration in regions where O_2_ supply is greatest, thereby allowing wider distribution of O_2_ to match metabolic demand (Hagen et al. 2003).

An improved spatial homogeneity of $\dot{Q}$O_2_ distribution relative to $\dot{V}$O_2_ demand, via its effect on increasing the mean capillary *P*O_2_ across active muscle sites, would be expected to enhance blood‐myocyte O_2_ flux and metabolic stability by raising intracellular *P*O_2_ (Hogan et al. 1992; McDonough et al. 2005), thereby potentially decreasing the $\dot{V}$O_2_ slow component ($\dot{V}$O_2Sc_) amplitude. Alternatively, an increased potential for O_2_‐independent NO bioavailability after BR supplementation, via its effect on increasing the number of red blood cells (RBCs) adjacent to active myocytes, may also attenuate the $\dot{V}$O_2Sc_ amplitude. For instance, the decreased $\dot{V}$O_2Sc_ following an initial bout of high‐intensity “priming” exercise has been reported to correlate inversely with an increased baseline total [Hb + Mb] across active muscle sites (Fukuoka et al. 2015). These findings suggest that development of the $\dot{V}$O_2Sc_ during high‐intensity constant work rate exercise (indicative of a progressive decline in efficiency as continuous high‐intensity exercise proceeds) is inversely linked to the skeletal muscle hyperemia and the associated improvement in muscle O_2_ diffusing capacity (D_m_O_2_) (Federspiel and Popel 1986; Groebe and Thews 1990). However, whilst the effect of BR supplementation on the $\dot{V}$O_2Sc_ amplitude is equivocal (Bailey et al. 2009; Kelly et al. 2013; Wylie et al. 2013), it has yet to be investigated whether the $\dot{V}$O_2Sc_ amplitude relates quantitatively to changes in total [Hb + Mb] across multiple muscle regions.

Using multi‐channel TRS‐NIRS, this study investigated the effect of NO_3_^−^‐rich BR supplementation on absolute deoxy [Hb + Mb] and total [Hb + Mb] responses within the *rectus femoris* (RF), *vastus lateralis* (VL), and *vastus medialis* (VM) during heavy‐intensity step cycling. We hypothesized that BR, which has been reported to increase skeletal muscle O_2_ delivery (Ferguson et al. 2013b) and lower skeletal muscle O_2_ utilization (Bailey et al. 2010) would: (1) slow the primary deoxy [Hb + Mb] mean response time (i.e. time delay + *τ*) and decrease the primary deoxy [Hb + Mb] amplitude, reflective of a lower fractional muscle O_2_ extraction; (2) reduce the spatial heterogeneity of these parameters resulting from a more uniform increase in O_2_ delivery relative to O_2_ utilization across the three locomotor muscles; and (3) decrease the $\dot{V}$O_2Sc_ amplitude in proportion with an increased total [Hb + Mb] across the three measurement sites.

## Methods

### Participants

Eight adult males (mean age = 24 ± 6 years; height = 175 ± 3 cm; body mass = 66 ± 5 kg) provided written informed consent to participate in this study, which was approved by *the Human Subjects Committee of Kobe Design University*, in compliance with *the Declaration of Helsinki*. All participants were physically active (mean peak $\dot{V}$O_2_ = 49.0 ± 6.6 mL^−1^ kg^−1^ min^−1^), were nonsmokers and were free of known cardiovascular, respiratory, and metabolic disease. Participants started the experimental procedure at least 2 h after their last meal having abstained from caffeine, alcohol, and intense exercise for 24 h before testing. The participants were also asked to refrain from using antibacterial mouthwash throughout the duration of the study (Govoni et al. 2008).

### Experimental protocol

The experiment was conducted in an environmental chamber (FLC‐2700S, Fuji Medical Science, Chiba, Japan) maintained at the ambient temperature of 21–24°C and relative humidity of 40–60%. All exercise tests were performed in the upright position on an electronically braked cycling ergometer (XL‐75III, Combi, Tokyo, Japan) with participants instructed to maintain pedal frequency at 60 rpm throughout exercise.

Participants were required to visit the laboratory on five occasions over a 4‐week period. On their first visit, participants completed a ramp incremental cycle test for the determination of peak $\dot{V}$O_2_ and gas exchange threshold (GET). The test included 2 min of rest, followed by 4 min of baseline pedaling at 20 W, and then ramp incremental cycling at 20 W min^−1^ to the limit of tolerance. The test was terminated when the subjects were unable to maintain 60 rev min^−1^ despite strong verbal encouragement. The peak $\dot{V}$O_2_ was taken as the highest 10‐s mean value attained before the subject's volitional exhaustion in the test. The GET was determined using the V‐slope method (Beaver et al. 1986) as the first disproportionate increase in CO_2_ production ($\dot{V}$CO_2_) relative to the increase in $\dot{V}$O_2_, and subsequently verified by an increase in the ventilatory equivalent for $\dot{V}$O_2_ (${\dot{V}}_{E}$ /$\dot{V}$O_2_) with no increase in ${\dot{V}}_{E}$ /$\dot{V}{\text{CO}}_{2}$. The work rates that would require 50% of the difference (Δ) between the GET and peak $\dot{V}$O_2_ (heavy‐intensity cycling, Δ50%) were subsequently determined, with account taken of the mean response time for $\dot{V}$O_2_ during ramp exercise (i.e. two thirds of the ramp rate was deducted from the work rate at the GET and peak$\dot{V}$O_2_, (Whipp et al. 1981).

Following the ramp test, participants were randomly assigned in a crossover, single‐blind design to receive 4 days supplementation with NO_3_^−^‐rich beetroot juice (BR) (140 mL/day; ~ 8.4 mmol NO_3_^−^; Beet It, James White Drinks, Ipswich, UK) or NO_3_^−^‐depleted BR juice as a placebo (PLA; 140 mL/day; 0.0034 mmol NO_3_^−^; Beet It, James White Drinks, Ipswich, UK). The PLA was identical in color, taste, smell and texture to the NO_3_^−^‐rich BR juice. The PLA beverage was created by passage of the juice, before pasteurization, through a column containing Purolite A520E ion exchange resin, which selectively removes NO_3_^−^ ions (Lansley et al. 2011). Four participants began with the BR condition, and the other four participants began with the PLA condition. The subjects were instructed to consume 70 mL BR in the morning and afternoon on *days 1* and *2* of the supplementation period. On *days 3* and *4*, the subjects were instructed to consume 140 mL BR over a 10‐min period, 2 h before the start of step exercise (see below), in accord with recent evidence that plasma [NO_2_^−^] peaks at approximately 2–2.5 h postadministration of BR containing 8.4 mmol of NO_3_^−^ (Wylie et al. 2013). A minimum of 7‐days washout separated each supplementation period. The subjects were also provided with a list of foods rich in nitrate (Hord et al. 2009) which was modified to include foods that are consumed frequently within ‘traditional' Japanese diet (Sobko et al. 2010) and asked to abstain from consuming these foods throughout the duration of the study.

On *days 3* and *4* of supplementation, the participants performed a single‐step exercise transition including 3‐min of ‘unloaded’ (20 W) baseline pedaling followed by 6‐min of heavy‐intensity cycling at an average power output of 197 ± 26 W. Before step exercise on *day 3*, venous blood samples were collected for the subsequent determination of plasma [NO_3_^−^] and [NO_2_^−^] (see Measurements below).

### Equipment and measurements

#### Plasma [NO_3_^−^] and [NO_2_^*−*^]

Venous blood samples (~4 mL) were drawn into lithium‐heparin tubes (7.5 mL Monovette Lithium Heparin, Sarstedt, Leicester, UK), which have very low levels of NO_2_^−^ and NO_3_^−^. Within 3 min of collection, the samples were centrifuged at 2700 g and 4°C for 10 min. Plasma was extracted and immediately frozen at −80°C for later analysis of [NO_2_^−^] and [NO_3_^−^] using a modification of the chemiluminescence technique (Bateman et al. 2002). All glassware, utensils, and surfaces were rinsed with deionized water to remove residual NO intermediates prior to [NO_2_^−^] and [NO_3_^−^] analysis. Plasma samples were deproteinized using zinc sulfate/sodium hydroxide precipitation prior to determination of [NO_3_^−^]. Firstly, 500 *μ*L of 0.18 N NaOH was added to 100 *μ*L of sample followed by 5 min incubation at room temperature. Subsequently, samples were treated with 300 *μ*L aqueous ZnSO4 (5% w/v) and vortexed for 30 sec before undergoing an additional 10 min incubation period at room temperature. Samples were then centrifuged at 2700 g for 5 min, and the supernatant was removed for subsequent analysis. The [NO_3_^−^] of the deproteinized plasma sample was determined by its reduction to NO in the presence of 0.8% (w/v) VCl_3_ in 1M HCl within an air‐tight purging vessel. Plasma samples were introduced to the vessel via 50 *μ*L injections into the septum at the top of the vessel. The spectral emission of electronically excited nitrogen dioxide, derived from the reaction of NO with ozone, was detected by a thermoelectrically cooled, red‐sensitive photomultiplier tube housed in a Sievers gas‐phase chemiluminescence nitric oxide analyzer (Sievers NOA 280i. Analytix Ltd, Durham, UK). The [NO_3_^−^] was determined by plotting signal (mV) area against a calibration plot of sodium nitrate standards. The [NO_2_^−^] of the undiluted (nondeproteinized) plasma was determined by its reduction to NO in the presence of glacial acetic acid and aqueous NaI (4% w/v) from sodium nitrite standards. 100 *μ*L injections were used for plasma [NO_2_^−^] determination.

#### Pulmonary O_2_ uptake

The breath‐by‐breath gas exchange measurement system (AE‐300S, Minato‐Medical, Osaka, Japan) was calibrated according to the manufacturer's recommendation before each exercise test. Subjects breathed through a low resistance, hot‐wire flowmeter for measurement of inspiratory and expiratory flow rates and volumes. Inspired and expired oxygen and carbon dioxide concentrations were continuously sampled from the mouth and measured by a gas analysis system. Gas volume and concentration signals were time‐aligned during gas analysis to account for the time lag between the signals.

#### Muscle deoxygenation

Absolute concentrations of oxygenated (oxy [Hb + Mb]), deoxygenated (deoxy [Hb + Mb]), and total (total [Hb + Mb]) hemoglobin and myoglobin were measured at three superficial quadriceps sites by two TRS‐20 NIRS machines (Hamamatsu photonics KK, Hamamatsu, Japan). Inter‐machine variability was <3% for measuring total [Hb + Mb] using optical phantoms. The accuracy of the TRS‐20 system has been described in previous studies (Hamaoka et al. 2000; Ijichi et al. 2005). The control system and measurement algorithm of this system has been described previously (Hamaoka et al. 2000; Ijichi et al. 2005; Chin et al. 2011; Koga et al. 2011; Spencer et al. 2014).

The skin under the probes was carefully shaved and the optodes housed in black rubber holders were fixed to the skin of the dominant thigh with adhesive tape. This helped minimize movement of the optodes during exercise. The interoptode spacing (OS) between irradiation and detection probes was 3 cm. The distal‐ region of *vastus lateralis* (VL) muscle and the mid‐belly of the *rectus femoris* (RF) and *vastus medialis* (VM) muscles were sampled on the same leg. After all the optodes were attached, a blackout curtain and bandage were wrapped around the leg to eliminate contamination from ambient light and improve the signal‐to‐noise ratio.

#### Ultrasonographic image

Doppler ultrasound (Logiq400, GE‐Yokogawa Medical Systems, Tokyo, Japan) was used to measure the depth of muscle and adipose tissue thickness (ATT) beneath the optode sites of each subject. This measurement was conducted with the subject seated in an upright position on a separate day from the exercise experiments. The Doppler images were collected with care to prevent pressure and distortion of the thickness of muscle and other tissues under the probe.

### Data analysis and kinetic modeling

The breath‐by‐breath $\dot{V}$O_2_ data from each step exercise transition were initially examined to exclude errant breaths by removing values lying more than four standard deviations from the local mean determined using a 5‐breath rolling average. Analysis of oxy [Hb + Mb], deoxy [Hb + Mb] and total [Hb + Mb] was performed following correction for ATT to a thickness of 0 mm using separate linear regression of the relationship between ATT and total [Hb + Mb] at rest (total [Hb + Mb] = −18.3 × [ATT] + 214, *r* ^2 ^= 0.57, *P *<* *0.001).

The filtered $\dot{V}$O_2_ and ATT‐corrected muscle deoxy [Hb + Mb] data were linearly interpolated and identical repetitions time aligned to the start of exercise and ensemble averaged into 5‐sec bins to improve the signal‐to‐noise ratio. The first 20 sec of $\dot{V}$O_2_ data after the onset of exercise was deleted to remove the phase I (cardio‐dynamic) response, and a mono‐exponential model with time delay was fitted to the averaged $\dot{V}$O_2_ data of the following form:

(1) $$\Delta Y_{\left(t\right)}=\Delta Y_{p}\left(1-e^{-(t-\mathrm{TD})/\tau p}\right)$$

where Δ*Y*_(t)_ indicates the value at a given time (*t*) minus the baseline value (60‐sec average) before exercise onset, Δ*Y*_*p*_ inidicates the amplitude change in the primary component from baseline to its asymptote, TD and *τ*_p_ represent the time delay and time constant of the primary exponential function, respectively. The model fitting window was constrained to exclude the $\dot{V}$O_2Sc_ and hence isolate the primary component using methods previously described (Rossiter et al. 2001). The parameter estimates from Eq. 1 were then resolved by least‐squares nonlinear regression (GraphPad Prism, GraphPad Software, San Diego, CA). The $\dot{V}$O_2Sc_ was determined by calculating the difference between the end‐exercise $\dot{V}$O_2_ and the sum of the primary amplitude and baseline $\dot{V}$O_2_. This was expressed both in absolute units and relative to end‐exercise $\dot{V}$O_2_ minus the O_2_ cost of baseline pedaling. For both conditions, the ‘gain’ of the primary $\dot{V}$O_2_ component (*G*_p_) was calculated by dividing the asymptotic primary amplitude by the increment in work rate (Δ$\dot{V}$O_2_/Δ*W*). To determine the overall $\dot{V}$O_2_ response dynamics (i.e. mean response time, MRT) during heavy‐intensity exercise, $\dot{V}$O_2_ data were also fit with a mono‐exponential model from *t* = 0 sec to end‐exercise, without TD.

The NIRS‐derived deoxy [Hb + Mb] response to exercise was also modeled to provide information on muscle (de)oxygenation. Since deoxy [Hb + Mb] increased after a short delay, the time onset for the exponential‐like rise in deoxy [Hb + Mb] was defined as the first datum lying > 1 SD above the mean baseline value (DeLorey et al. 2003). Subsequently, the deoxy [Hb + Mb] data were fit to 180 sec using Equation (1) after omitting data points preceding the exponential‐like increase. The TD and *τ*_p_ of the deoxy [Hb + Mb] response was then summed to reflect the primary MRT (MRT_p_) during the initial 180 sec of exercise. Total [Hb + Mb] responses do not approximate an exponential and were therefore not modeled; however, we did estimate the area under the curve (AUC) to provide an estimate of blood volume changes by integrating between the measured response during exercise and the pretransition baseline value.

### Statistical analyses

Data are presented as mean ± SD. Paired‐samples *t*‐tests were used to compare the $\dot{V}$O_2_ kinetic parameters between PLA and BR. Intersite coefficient of variation [CV (%); 100·SD/mean of the three sites values] was calculated for each subject to show spatial heterogeneity of quadriceps deoxygenation. A two‐way, treatment (PLA and BR) × muscle site (RF and VL and VM) repeated‐measures ANOVA was used to explore differences in deoxy [Hb + Mb] and total [Hb + Mb] responses. Significant effects were further explored using Fisher's LSD post hoc *t*‐tests. Effect size (ES; using Cohen's *d*) and statistical power (1‐*β*) were also calculated for comparison of $\dot{V}$O_2_ and NIRS‐derived variables. Pearson product‐moment correlation coefficients (*r*) were used to investigate the relationship between outcome variables. Statistical significance was accepted when *P *<* *0.05.

## Results

### Plasma [nitrate] and [nitrite]

The plasma [NO_3_^−^] on day 3 of supplementation was significantly increased following BR compared to PLA (600 ± 73 vs. 21 ± 6 *μ*mol/L, *P *<* *0.001). Likewise, plasma [NO_2_^−^] on day 3 was elevated following BR compared to PLA (321 ± 122 vs. 130 ± 23 nmol/L, *P *=* *0.003).

### Pulmonary O_2_ uptake kinetics

**Figure 1:**
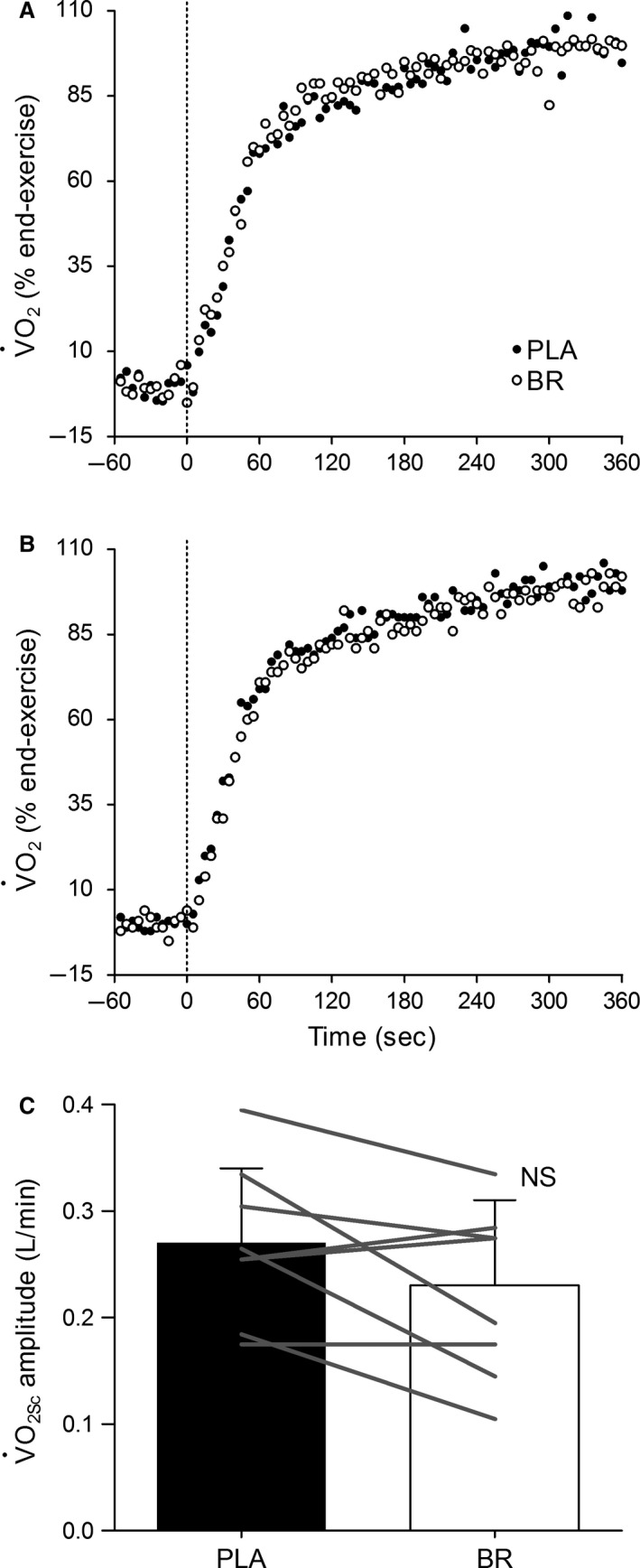
Pulmonary oxygen uptake ($\dot{V}$O_2_) profiles in two individual subjects (A and B) during heavy‐intensity step exercise. The *y*‐axis values are normalized relative to end‐exercise to facilitate comparison between PLA (*closed circles*) and BR (*open circles*) conditions. The onset of step exercise is indicated by the vertical dotted line. Panel A: BR speeded (~12%) the $\dot{V}$O_2_ mean response time consequent to a decreased $\dot{V}$O_2_ slow component ($\dot{V}$O_2Sc_) amplitude in a “responder” to nitrate supplementation. Panel B: There were no differences in $\dot{V}$O_2_ kinetics between supplement conditions for a “non‐responder” subject. Panel C: Mean ± SD $\dot{V}$o_2Sc_ amplitude with individual subject data superimposed (solid gray lines). NS, not significant (*P *=* *0.07) between supplement conditions.

The group mean parameters of $\dot{V}$O_2_ kinetics are presented in Table 1 with the $\dot{V}$O_2_ response from two individual subjects shown in Figure 1A and B. There were no significant differences between PLA and BR in the primary $\dot{V}$O_2_ amplitude or gain throughout the fundamental phase (*P *>* *0.16). The $\dot{V}$O_2Sc_ amplitude decreased in 5/8 subjects in BR compared to PLA (Fig. 1C); however, mean differences were not significant between supplement conditions expressed in absolute units (*P *=* *0.07, ES = 0.5, 1‐*β *= 0.33) or when normalized relative to the total Δ$\dot{V}$O_2_ above baseline pedaling (PLA: 14 ± 4 vs. BR: 11 ± 4 %, *P *=* *0.08, ES = 0.8, 1‐*β *= 0.56). Modeling the entire $\dot{V}$O_2_ response from *t *=* *0 sec to end‐exercise yielded no significant differences in the overall $\dot{V}$O_2_ MRT between PLA and BR (*P *=* *0.56). Likewise, the percentage of peak $\dot{V}$O_2_ attained at end‐exercise was not different following PLA and BR supplementation (82 ± 6 vs. 82 ± 9 %, *P *=* *0.84).

**Table 1:** Kinetics of pulmonary O_2_ uptake ($\dot{V}$O_2_) during heavy‐intensity step exercise following PLA and BR supplementation

	PLA	BR
BL (L min^−1^)	0.58 ± 0.04	0.59 ± 0.07
*τ*_p_ (sec)	26 ± 5	30 ± 8
*A*_p_ (L min^−1^)	1.73 ± 0.28	1.77 ± 0.20
*G*_p_ (mL min^−1^ W^−1^)	9.8 ± 0.5	10.1 ± 0.6
TD_Sc_ (sec)	155 ± 20	177 ± 20
*A*_Sc_ (L min^−1^)	0.27 ± 0.07	0.23 ± 0.08
*Relative A_Sc_* (%)	14 ± 4	11 ± 4
End $\dot{V}$O_2_	2.58 ± 0.29	2.58 ± 0.25
MRT (sec)	58 ± 9	56 ± 13

Values are mean ± SD. BL, baseline; *τ*_p_, time constant of phase II; *A*_p_, amplitude of phase I + phase II, not including BL; TD_Sc_, time delay of slow component; *A*_Sc_, amplitude of slow component; MRT, $\dot{V}$O_2_ mean response time. No significant differences (*P *>* *0.05) between supplement conditions.

### Absolute deoxy [Hb + Mb] and total [Hb + Mb] responses

**Figure 2:**
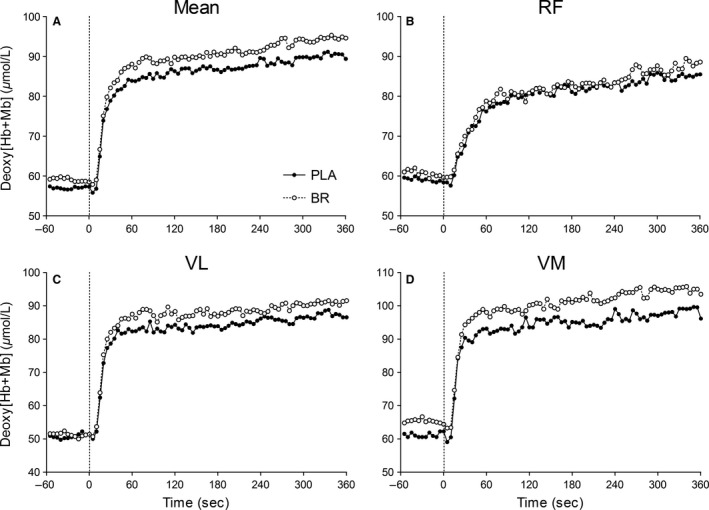
Mean ATT‐ corrected absolute deoxy [Hb + Mb] across the three measurement sites within the *rectus femoris* (RF), *vastus lateralis* (VL), and *vastus medialis* (VM) (Panels A–D, respectively) during heavy‐intensity step exercise following PLA (*closed circles*) and BR supplementation (*open circles*). Please note error bars are excluded for clarity. The onset of step exercise is indicated by the vertical dotted line.

The kinetics and amplitude of deoxy [Hb + Mb] within each muscle are shown in Table 2 with corresponding mean profiles illustrated in Figure 2. The deoxy [Hb + Mb] TD and *τ*_p_ increased within the RF compared to other muscles following exercise onset. Consequently, there was a significant main effect (*P *=* *0.02) for muscle region on the deoxy [Hb + Mb] MRT_p_ which was slower for RF compared to VL and VM (*P *≤* *0.03, 1−*β *= 1.00). There was no significant main effect (*P *>* *0.15) for supplement condition on the deoxy [Hb + Mb] MRT_p_ or *A*_p_ following exercise onset.

**Table 2:** Kinetics and amplitude of absolute deoxy [Hb + Mb] within the *rectus femoris* (RF), *vastus lateralis* (VL), and *vastus medialis* (VM) during heavy‐intensity step exercise following PLA and BR supplementation

	PLA			BR		
	RF	VL	VM	RF	VL	VM
BL (*μ*mol/L)	59 ± 7	51 ± 5^1^	61 ± 8^2^	61 ± 5	51 ± 4^1^	65 ± 10^2^
*A*_p_ (*μ*mol/L)	24 ± 8	33 ± 12	33 ± 11	24 ± 9	36 ± 14	34 ± 9
End (*μ*mol/L)	85 ± 10	87 ± 15	99 ± 10^1^	88 ± 9	91 ± 16	105 ± 17^1^ ^,^ ^2^
TD (sec)	17 ± 7	9 ± 4	11 ± 2	19 ± 10	9 ± 3	11 ± 2
*τ*_p_ (sec)	45 ± 44	11 ± 4	9 ± 3	37 ± 38	13 ± 6	11 ± 4
MRT_p_ (sec)	62 ± 42	20 ± 7^1^	20 ± 3^1^	56 ± 35	22 ± 7^1^	22 ± 4^1^

Values are mean ± SD. deoxy [Hb + Mb], absolute concentration of deoxyhemoglobin + myoglobin; *A*_p_, amplitude of primary component; TD, time delay of the initial deoxy [Hb + Mb] increase; *τ*_p_, time constant of the primary component; MRT_p_, mean response time of the primary component (TD + *τ*_p_). Significant differences (*P *<* *0.05) vs.

^1^RF and versus ^2^VL. No significant differences (*P *>* *0.05) between supplement conditions.

The kinetics and amplitude of deoxy [Hb + Mb] across the three measurement sites and their intersite CV are shown in Tables 3 and 4, respectively. There were no significant differences in the muscle spatial heterogeneity of the deoxy [Hb + Mb] MRT_p_ or *A*_p_ between PLA and BR supplementation (*P *>* *0.3). Compared to PLA, BR tended to increase the absolute primary deoxy [Hb + Mb] amplitude (i.e. *A*_a* *_= BL + *A*_p_) across the three measurement sites (*P *=* *0.07, ES = 0.4, 1‐*β *= 0.24). Consequently, the mean (three‐site) deoxy [Hb + Mb] at end‐exercise was significantly increased in BR compared to PLA (*P *=* *0.04, ES = 0.4, 1−*β *= 0.19; Table 4, Fig. 2A).

**Table 3:** Kinetics of absolute deoxy [Hb + Mb] across the three measurement sites during heavy‐intensity step exercise following PLA and BR supplementation

	PLA	BR
TD (sec)	12 ± 2	13 ± 4
*τ*_p_ (sec)	22 ± 15	20 ± 14
MRT_p_ (sec)	34 ± 14	33 ± 13
CV of TD (%)	32 ± 35	38 ± 26
CV of *τ*_p_ (%)	77 ± 37	60 ± 31
CV of MRT_p_ (%)	62 ± 35	55 ± 27

Values are mean ± SD. CV, muscle intersite coefficient of variation. No significant differences (*P* > 0.05) between supplement conditions.

**Table 4:** Amplitude of absolute deoxy [Hb + Mb] across the three measurement sites during heavy‐intensity step exercise following PLA and BR supplementation

	PLA	BR
BL (*μ*mol/L)	57 ± 5	59 ± 4
*A*_p_ (*μ*mol/L)	30 ± 7	32 ± 8
*A*_a_ (*μ*mol/L)	87 ± 8	91 ± 10
End (*μ*mol/L)	91 ± 9	95 ± 12^1^
CV of BL (%)	12 ± 7	14 ± 8
CV of *A*_p_ (%)	32 ± 15	30 ± 14

Values are mean ± SD. *A*_a_, absolute amplitude of primary component (BL + *A*_p_).

^1^ *P *<* *0.05 vs. PLA condition.

**Figure 3:**
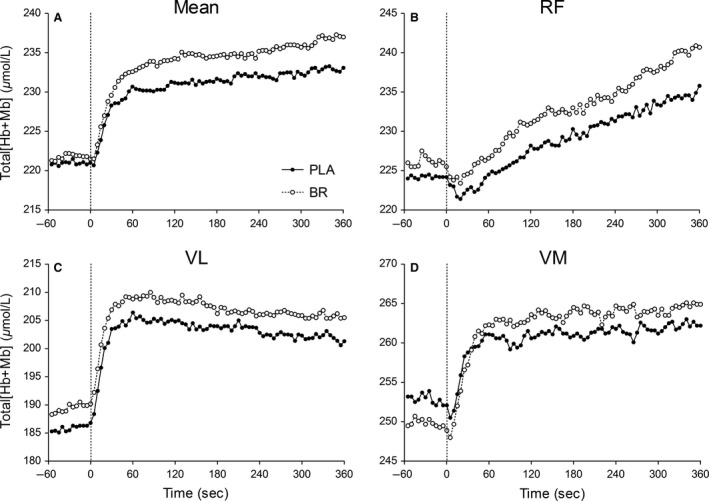
Mean ATT‐ corrected absolute total [Hb + Mb] across the three measurement sites and within the *rectus femoris* (RF), *vastus lateralis* (VL), and *vastus medialis* (VM) (Panels A–D, respectively) during heavy‐intensity step exercise following PLA (*closed circles*) and BR supplementation (*open circles*). Please note error bars are excluded for clarity. The onset of step exercise is indicated by the vertical dotted line.

Total [Hb + Mb] responses within each muscle are presented in Figure 3. There was substantial variation in baseline total [Hb + Mb] with differences significant between individual muscles (*P *≤* *0.03). There was no significant main effect (*P *>* *0.18) for supplement condition on total [Hb + Mb] at baseline or at end‐exercise. Compared to PLA, BR tended to increase the AUC for total [Hb + Mb] across the three measurement sites (PLA: 3650 ± 1188 vs. BR: 4467 ± 1315 *μ*mol/L sec^−1^, *P *=* *0.08, ES = 0.7, 1−*β *= 0.50; Fig. 3A).

### Relationships between peak aerobic fitness, NO biomarkers, $\dot{V}$o_2_ kinetics and muscle oxygenation

**Figure 4:**
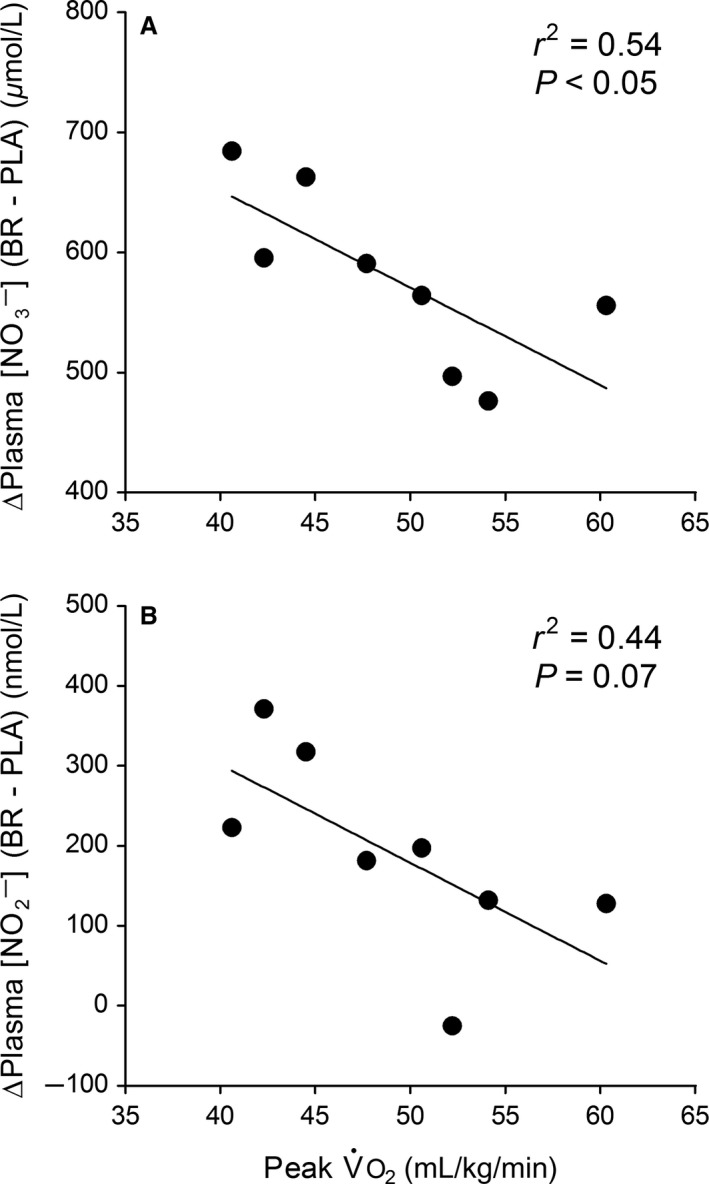
Relationship between the BR‐induced increase (Δ) in plasma [NO_3_^−^] and [NO_2_^−^] on day 3 of supplementation with peak $\dot{V}$O_2_ during ramp incremental cycling.

**Figure 5:**
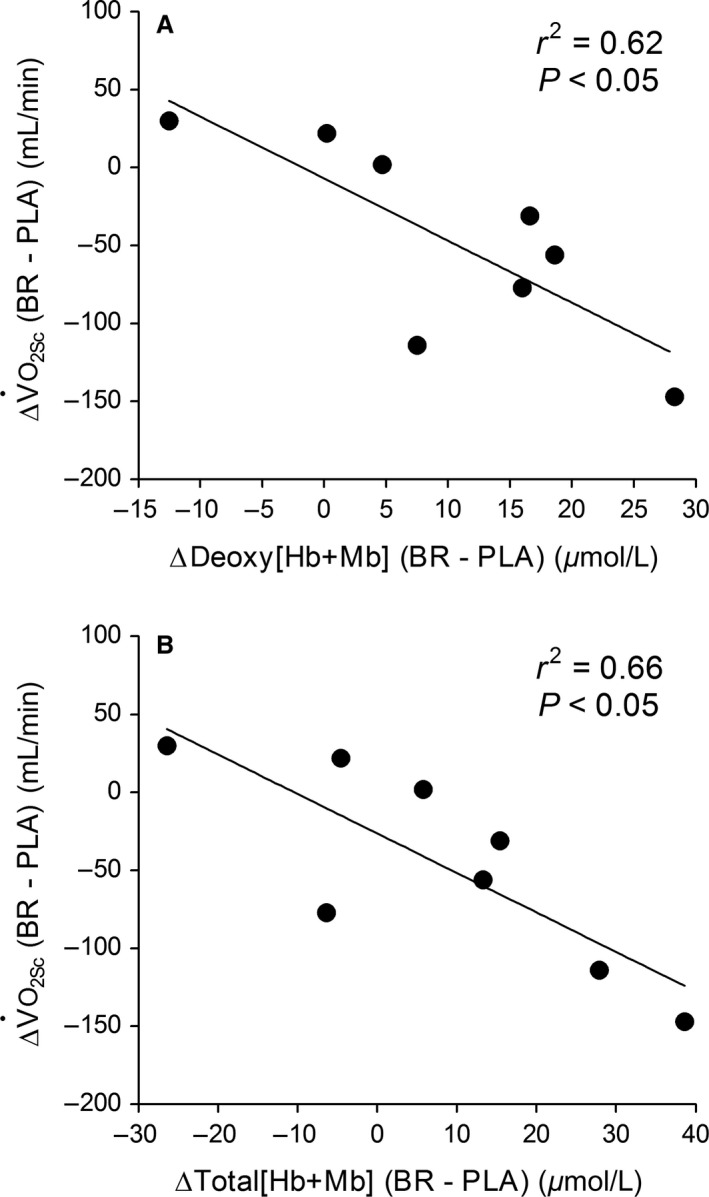
Relationship between the BR‐induced reduction of the $\dot{V}$O_2Sc_ amplitude with absolute differences in total [Hb + Mb] and deoxy [Hb + Mb] across the three measurement sites. Values on the *x*‐axis represent the sum of differences between BR minus PLA at baseline, 180 sec and 360 sec during heavy‐intensity step exercise.

There was an inverse relationship between peak $\dot{V}$O_2_ (mL kg^−1^ min^−1^) during the initial ramp test with the increase (Δ) in plasma [NO_3_^−^] and [NO_2_^−^] following BR compared to PLA supplementation (*r* ^2^ = 0.54 and 0.44, *P *=* *0.04 and 0.07, respectively; Fig. 4). The BR‐induced $\dot{V}$O_2Sc_ reduction (*P *=* *0.07) was inversely correlated with differences (summed) in total [Hb + Mb] and deoxy [Hb + Mb] across the three measurement sites during heavy‐intensity step cycling (*r* ^2^ = 0.66 and 0.62, *P *<* *0.021; Fig. 5).

## Discussion

This is the first study to investigate the effect of inorganic NO_3_^−^ supplementation on absolute [Hb + Mb] responses within multiple locomotor muscles during exercise. The principal original finding was that NO_3_^−^‐rich beetroot juice supplementation did not alter the muscle intersite CV of the primary deoxy [Hb + Mb] mean response time or amplitude following the onset of heavy‐intensity step cycling. There were no significant differences in primary $\dot{V}$O_2_ kinetics between supplement conditions; however, NO_3_^−^‐rich beetroot juice supplementation decreased the $\dot{V}$O_2Sc_ in proportion with increases in deoxy [Hb + Mb] and total [Hb + Mb] across the *rectus femoris, vastus lateralis, and vastus medialis*. These findings suggest that NO_3_^−^ treatment does not alter the spatial heterogeneity of the dynamic balance between muscle O_2_ delivery relative to O_2_ utilization during heavy submaximal exercise. Rather, NO_3_^−^ supplementation increased the capacity for muscle diffusive O_2_ transport across active muscle sites, as reflected by higher end‐exercise deoxy [Hb + Mb] and total [Hb + Mb], which correlated with a lower $\dot{V}$O_2Sc_ amplitude. These findings contribute to our understanding of the mechanisms by which short‐term NO_3_^−^ supplementation can improve efficiency during continuous submaximal exercise (Larsen et al. 2007; Bailey et al. 2010).

### Effect of BR supplementation on plasma [NO_3_^−^] and [NO_2_^−^]

There was a significant increase in plasma [NO_2_^−^] following BR compared to PLA supplementation; however, the magnitude (Δ) of this increase (~191 nmol/L) was lower compared to previous studies in which an equivalent NO_3_^−^ dose was administered acutely (~291 nmol/L) (Wylie et al. 2013) or following 4–6 days of supplementation (~283–340 nmol/L) (Breese et al. 2013; Kelly et al. 2014). An important difference is the study population used in the present investigation, which consisted of Japanese natives. It has been reported that ‘traditional’ foods contained within a Japanese diet are high in NO_3_^−^ (Sobko et al. 2010). Therefore, whilst requesting that our subjects refrain from consuming these foods throughout the study period, it is conceivable that chronic exposure to a NO_3_^−^‐rich diet may have mitigated the influence of BR supplementation on increasing biomarkers of NO synthesis. In turn, this may explain the lack of significant effects on $\dot{V}$O_2_ kinetics in this study compared to others (Bailey et al. 2009, 2015; Lansley et al. 2011; Breese et al. 2013).

We also reported an inverse relationship between the BR‐induced increase in plasma [NO_3_^−^] and [NO_2_^−^] with subjects' peak $\dot{V}$O_2_ (mL kg^−1^ min^−1^) during ramp incremental cycling (Fig. 4). It has been reported that exercise training increases endothelial NO synthase‐ (eNOS) dependent NO production (Lauer et al. 2001), which may explain higher endogenous levels of plasma [NO_3_^−^] and [NO_2_^−^] as metabolites of NO oxidation in the aerobically trained state (Totzeck et al. 2012). Consequently, in this study, exogenous supplementation with NO_3_^−^ may have had a less pronounced effect on increasing the potential for O_2_‐independent NO synthesis in subjects with higher initial peak aerobic fitness.

### Effect of BR supplementation on the spatial heterogeneity of quadriceps deoxy [Hb + Mb] responses

There were no significant differences between PLA and BR in the deoxy [Hb + Mb] MRT_p_ or *A*_p_ within the RF, VL, and VM muscles following the onset of heavy‐intensity step cycling. A previous investigation reported that 6‐days of BR supplementation lowered the deoxy [Hb + Mb] *A*_p_ within the VL during moderate‐intensity cycling (Bailey et al. 2009); however, it has been reported that NO_3_^−^‐rich BR does not alter deoxy [Hb + Mb] responses within this muscle site during severe‐intensity cycling transitions elicited from unloaded pedaling (Kelly et al. 2014). An important limitation from previous studies using CW‐NIRS is the assumption of constant tissue optical properties, which has been shown to inaccurately estimate muscle deoxy [Hb + Mb] signals due to increased light scattering (Ferreira et al. 2007). Therefore, an important novel finding using TRS‐NIRS was that NO_3_^−^‐rich BR supplementation did not alter the rate or amplitude of deoxy [Hb + Mb] within the VL during which changes in light path length, absorption and scattering coefficients were corrected for.

An additional novel feature of this study was the application of TRS‐NIRS within an increased number of active muscle sites (other than the VL) following PLA and BR supplementation. In line with previous studies (Koga et al. 2007, 2011; Chin et al. 2011; Fukuoka et al. 2015), this revealed inter‐muscular differences in deoxy [Hb + Mb] profiles which may be explained by disparities in muscle fiber type (Johnson et al. 1973; Lexell et al. 1983) and/or activity level (Chin et al. 2011) between skeletal muscles. However, there were no significant differences in the inter‐site CV of the deoxy [Hb + Mb] MRT_p_ or *A*_p_ between PLA and BR supplementation. Therefore, by extension, NO_3_^−^ treatment did not alter the muscle spatial heterogeneity in $\dot{Q}$O_2_‐to‐$\dot{V}$O_2_ matching during heavy‐intensity step cycling.

Although deoxy [Hb + Mb] dynamics were not different between the BR and PLA trials at the level of individual muscles, we did find a significant increase in the mean (three‐site) end‐exercise doxy [Hb + Mb] following BR compared to PLA supplementation (Fig. 2A; Table 4). It has been reported that BR supplementation increases locomotor muscle blood flow (Ferguson et al. 2013b) and slows microvascular *P*O_2_ kinetics (i.e., increased microvascular *P*O_2_ across the on‐exercise transition) during stimulated contractions in animal muscle (Ferguson et al. 2013a). Since deoxy [Hb + Mb] exhibits similar response dynamics to that of microvascular *P*O_2_ following the onset of skeletal muscle contractions (Koga et al. 2012), and hence reflects the dynamic balance between local O_2_ delivery to O_2_ utilization, we hypothesized that BR ingestion would slow the rate (i.e. MRT_p_) and reduce the amplitude of deoxy [Hb + Mb] kinetics during voluntary exercise in humans. However, in this study, for the same $\dot{V}$O_2_ dynamics between supplement conditions, an increased end‐exercise deoxy [Hb + Mb] with BR suggests NO_3_^−^ supplementation increased proportionally muscle fractional O_2_ extraction on transition to a higher metabolic rate.

The AUC for total [Hb + Mb] (hence microvascular blood volume) across the three measurement sites increased following BR compared to PLA supplementation with a moderate‐to‐large effect size (Fig. 3A). This response in the BR trial may have reflected the effect of an increased plasma [NO_2_^−^] per se (Gladwin et al. 2000) or other reactive nitrogen intermediates generated from NO_3_^−^ treatment (Pinheiro et al. 2016) on augmenting the vascular response following the onset of step exercise. Accordingly, an increased number/volume of RBCs by longitudinal capillary recruitment (increased hematocrit, Poole et al. 2013), hence D_m_O_2_ (Federspiel and Popel 1986; Groebe and Thews 1990), would be expected to have facilitated O_2_ release from capillaries, which may explain the increased muscle fractional O_2_ extraction reported herein following NO_3_^−^ supplementation.

### Effect of BR supplementation on O_2_ uptake kinetics

There were no significant differences in the $\dot{V}$O_2_ *τ*_p_ following BR compared to PLA supplementation, which is consistent with previous studies during high‐intensity step cycling in normoxia at mid‐range pedal rates (Bailey et al. 2009; Kelly et al. 2013, 2014). It has been reported that NO_3_^−^ treatment speeds O_2_ uptake kinetics when the recruitment of higher‐order type II muscle fibers is experimentally manipulated in humans (Jones et al. 2016). For example, BR intake has been reported to speed the $\dot{V}$O_2_ *τ*_p_ during cycling at fast pedal cadences (~115 rpm) (Bailey et al. 2015) and when transitioning from a raised baseline work rate (Breese et al. 2013). Therefore, whilst heavy‐intensity cycling transitions at ~60 rpm elicited from unloaded pedaling would have been expected to recruit a population of higher‐order type II muscle fibers (Gollnick et al. 1974; Vollestad and Blom 1985; Krustrup et al. 2004), their proportional contribution to the power production may not have increased sufficiently in the present investigation; hence, this may explain the lack of significant effects on the $\dot{V}$O_2_ *τ*_p_ following BR supplementation.

Whilst not significantly different between BR and PLA supplementation, there were individual ‘responders’ whom lowered their $\dot{V}$O_2Sc_ amplitude following NO_3_^−^ treatment (Fig. 2A). Moreover, the relative $\dot{V}$O_2Sc_ amplitude decreased in BR compared to PLA with a large effect size. We also reported a significant inverse correlation between the $\dot{V}$O_2Sc_ reduction in the BR compared to the PL trial with increases in total [Hb + Mb] and deoxy [Hb + Mb] across the three measurement sites (Fig. 5). In accord with Fick's law [$\dot{V}$O_2 _= D_m_O_2_(P_capillary_O_2_–P_intramyocyte_O_2_)], an increased microvascular blood volume (hence D_m_O_2_, Federspiel and Popel 1986; Groebe and Thews 1990) would permit $\dot{V}$O_2_ to increase with less reduction in P_intramyocyte_O_2_, thereby “tightening” mitochondrial control (i.e. decreasing ∆[PCr], ∆[Pi], ∆[NADH], and ∆[ADP]free, Hogan et al. 1992) and potentially decreasing the $\dot{V}$O_2Sc_ amplitude. In line with this proposal, a previous study (Fukuoka et al. 2015) reported that “priming” exercise decreased the $\dot{V}$O_2Sc_ in proportion with an increased baseline total [Hb + Mb] across the RF and VL muscles. Therefore, our findings support that individual differences in decreasing of the $\dot{V}$O_2Sc_ amplitude with NO_3_^−^ relate quantitatively to increases in diffusive conductance (i.e. transmembrane O_2_ flux/O_2_ extraction) across active muscle sites.

### Methodological limitations

It is recognized that the absorbance signals of both chromophores (i.e. Hb and Mb) overlap in the near‐infrared region (i.e. 650–900 nm); therefore, desaturation of Mb may have contributed significantly to the NIRS‐derived muscle deoxygenation data (Masuda et al. 2010). In addition, the output power delivered at the extremity of the NIRS irradiation optical fiber was 100 *μ*W with interoptode spacing of 3 cm at each muscle site. Consequently, in this study, deoxy [Hb + Mb] measurement was localized to ~1.5 cm in depth; therefore, we were unable to resolve the extent to which BR supplementation altered the balance between $\dot{Q}$O_2_‐to‐$\dot{V}$O_2_ across the muscle thickness. Finally, the relatively low statistical power (1‐*β*) may have increased the potential for type II error; in particular, with regard to detecting significant differences in the mean $\dot{V}$O_2Sc_ amplitude between PLA and BR conditions.

## Conclusions

Short‐term supplementation with BR juice increased plasma [NO_2_^−^] and thus the potential for O_2_‐independent NO synthesis in this study. However, compared to the PLA condition, there were no significant differences in the spatial variance of absolute deoxy [Hb + Mb] kinetics across the RF, VL, and VM muscles following the onset of heavy‐intensity step cycling. However, BR supplementation significantly increased the mean (three‐site) end‐exercise deoxy [Hb + Mb] during which total [Hb + Mb] was augmented throughout exercise. Since $\dot{V}$O_2_ kinetics was not different between the BR and PLA trials, this suggests NO_3_^−^ treatment increased muscle O_2_ diffusive capacity during heavy‐intensity cycling transitions. Importantly, the $\dot{V}$O_2Sc_ reduction in BR compared to PLA, whilst not significant, was inversely associated with increases in deoxy [Hb + Mb] and total [Hb + Mb] across the three muscle regions. Therefore, the findings of this study improve understanding of the mechanisms by which NO_3_^−^ supplementation might improve efficiency during continuous submaximal exercise.

## Conflict of Interest

None declared.
